# Hidradenitis Suppurativa: A Perspective on Genetic Factors Involved in the Disease

**DOI:** 10.3390/biomedicines10082039

**Published:** 2022-08-21

**Authors:** Chiara Moltrasio, Paola Maura Tricarico, Maurizio Romagnuolo, Angelo Valerio Marzano, Sergio Crovella

**Affiliations:** 1 Dermatology Unit, Fondazione IRCCS Ca’ Granda Ospedale Maggiore Policlinico, 20122 Milan, Italy; 2 Department of Medical Surgical and Health Sciences, University of Trieste, 34137 Trieste, Italy; 3 Institute for Maternal and Child Health IRCCS Burlo Garofolo, 34137 Trieste, Italy; 4 Department of Pathophysiology and Transplantation, Università degli Studi di Milano, 20122 Milan, Italy; 5 Biological Science Program, Department of Biological and Environmental Sciences, College of Arts and Sciences, Qatar University, Doha P.O. Box 2713, Qatar

**Keywords:** hidradenitis suppurativa, genetic disease, dermatological disease, acne inversa

## Abstract

Hidradenitis Suppurativa (HS) is a chronic inflammatory skin disease of the pilosebaceous unit, clinically consisting of painful nodules, abscesses, and sinus tracts mostly in, but not limited to, intertriginous skin areas. HS can be defined as a complex skin disease with multifactorial etiologies, including—among others—genetic, immunologic, epigenetic, and environmental factors. Based on genetic heterogeneity and complexity, three different forms can be recognized and considered separately as sporadic, familial, and syndromic. To date, several genetic variants associated to disease susceptibility, disease-onset, and/or treatment response have been reported; some of these reside in genes encoding the gamma-secretase subunits whereas others involve autoinflammatory and/or keratinization genes. The aim of this perspective work is to provide an overview of the contribution of several genetic studies encompassing family linkage analyses, target candidate gene studies, and -omic studies in this field. In our viewpoint, we discuss the role of genetics in Hidradenitis suppurativa considering findings based on Sanger sequencing as well as the more recent Next Generation Sequencing (i.e., exome sequencing or RNA Sequencing) with the aim of better understanding the etio-pathogenesis of the disease as well as identifying novel therapeutic strategies.

## 1. Introduction

Hidradenitis suppurativa (HS), also known as acne inversa, is a chronic inflammatory skin disease involving the pilo-sebaceous unit with a prevalence in Europe of 0.8%, ranging from 0.5 to 1.3% [1].

Risk factors, particularly a predisposing genetic background, obesity, smoking, and skin occlusion have been strongly linked to HS onset, suggesting that HS could be the result of the combined environmental/lifestyle factors in a genetically predisposed individual, although the mechanisms to which the crosstalk between genetic and non-genetic factors determine disease phenotype as well as response to therapy are not fully understood [2].

**Figure 1:**
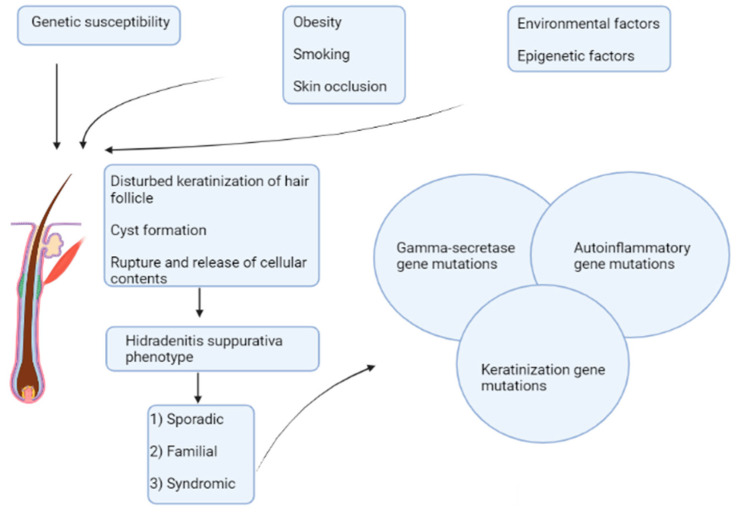
Schematic representation of the three different forms of HS, based on its multifactorial aetiologies and genetic heterogeneity.

Currently, based on genetic heterogeneity and complexity, three different forms of HS can be recognized: familial, sporadic, and syndromic forms (Figure 1).

In Western countries, HS usually presents as a sporadic form but in 40% of cases may occur as a familial disorder (OMIM:# 142690 ACNE INVERSA, FAMILIAL, 1; ACNINV1 and # 613736 ACNE INVERSA, FAMILIAL, 2, ACNINV2) [2]; in addition to its occurrence as a single disease, HS can be considered as a common clinical manifestation in the setting of other immune-mediated inflammatory diseases or inherited disorders, thus presenting as syndromic HS [3], whose prototype is represented by the clinical triad of Pyoderma gangrenosum (PG), acne, and suppurative hidradenitis (PASH). The other main autoinflammatory syndromes involving HS are the following: PASH with pyoderma gangrenosum (PG), namely PAPASH; psoriatic arthritis, PG, acne and suppurative hidradenitis (PsAPASH); pustular psoriasis, arthritis, PG, synovitis, acne and suppurative hidradenitis (PsAPSASH), and PG, acne, suppurative hidradenitis, and ankylosing spondylitis (PASS) [4]; moreover, we also have to consider the syndrome of synovitis, acne, pustulosis, hyperostosis, and osteitis (SAPHO), which incorporates a combination of cutaneous and articular symptoms including HS [5]; overall, the above-mentioned HS syndromic forms are very rare conditions and the exact prevalence has not yet been reported.

A great number of genetic studies, and the increasing availability of -omic approaches in both clinical and research settings and the support of in vitro and in vivo functional studies—although these latter to a lesser extent—contributed to elucidating some pathogenetic mechanisms and the causative role of certain variants driving HS susceptibility and disease onset; however, further efforts in this direction are mandatory to fully elucidate the immunologic and genetic landscape of HS and its forms.

Here, we present our perspective considering the more relevant genetic findings on HS and its syndromic forms, all characterized by great genetic heterogeneity (Table 1), highlighting the contribution of each study to unravel the complex genetic puzzle that plays an important role in the etiopathogenesis of HS or HS-associated diseases.

## 2. Clinical Features of Hidradenitis Suppurativa

HS typical lesions are deep-seated painful nodules, abscesses, and draining sinus tracts (fistulas) which can lead to extensive bridged or mesh-like scarring and double-ended open pseudocomedones in apocrine-glands bearing regions of the body, namely in the inguinal folds, axillae, intermammary, and anogenital areas; the lesions are usually chronic-recurrent with 2–3 episodes of inflammation over 6 months [26], leading to a high extent of physical distress, with social embarrassment and depression, and with the most severe impairment of life quality among common dermatoses [27].

In the attempt to categorize HS phenotypes, different scoring systems have been proposed to assess disease severity; the first by Hurley in 1989 [28], who classified HS in three stages (mild—moderate—severe) based on the lesions’ extent and severity [28], as well as the Physician global assessment (PGA), a six-stage system (from clear to very severe) depending on the number of the lesions [29]. Recently, Zouboulis et al. [30] validated the dynamic IHS4 (International Hidradenitis Suppurativa Severity Score System) score; the final IHS4 score (points) corresponds to number of nodules multiplied by 1 + number of abscesses multiplied by 2 + number of draining tunnels (fistulae/sinuses) multiplied by 4 [30]; a score of 3 or less corresponds to mild HS, a score of 4–10 signifies moderate HS, and a score of 11 or higher implies severe HS [30]. The IHS4 score system represents a quick and useful tool for assessing disease severity both in clinical trials and in real-life, and to monitor clinical and treatment outcomes [30].

The above-mentioned scoring systems, however, could not reflect the wider clinical spectrum of HS and the associated comorbidities, especially in the syndromic forms; for these reasons, considering the clinical variability of the disease and the lack of a homogeneous classification, Van der Zee et al. [28] proposed to classify HS in six clinical phenotypes: (i) regular type, which represents the typical lesions described above, (ii) frictional furuncle type, characterized by nodules and abscesses in friction areas (buttocks, legs, and abdomen), which rarely evolve into fistulas, (iii) scarring folliculitis type, in which classical superficial lesions (Hurley stage I) are frequently followed by cribriform scarring and pseudocomedones, (iv) conglobate type, in which cyst formation and acne conglobata predominates on the face and upper back with a strong familiar history, (v) syndromic type in patients with concomitant diseases such as inflammatory bowel disease (IBD), arthritis, and autoinflammatory syndromes, and finally, (vi) ectopic type, in which the main area involved is the face [30]. Subsequently, Dudnik et al. [31], based on real-life observation on a Dutch cohort of patients, suggested that the ectopic and syndromic phenotypes were not specific, lacking distinctive clinical features and could be categorized as one of the other phenotypes; interestingly, a positive family history did not differ between the phenotypes. Frew et al. [32] assessed the inter-rater reliability of HS phenotypes described in the literature and based on genotype-phenotype correlation and proposed a revision of the classification limiting it into a: (i) typical HS, corresponding to the regular type, (ii) atypical HS, including scarring folliculitis and conglobate types, and (iii) syndromic HS [32].

## 3. Therapeutic Strategies for HS

HS therapeutic options are based on disease severity and include a combination of topical and systemic therapies, including biologic drugs, surgical approach, as well as general measures and analgesics to reduce both pain and the burden of disease [33]. Topical application of clindamycin twice a day on the affected areas represents the mainstay treatment in the milder localized form of HS, whereas systemic antibiotic therapies with tetracyclines, clindamycin, and/or rifampicin in different regimens is often required to control the disease and reduce the flares in non-responders to topical therapy and moderate HS [26]; acitretin and dapsone could be employed as an alternative to, or in combination with, antibiotic therapy in mild-to-moderate HS [26]. Adalimumab, an antagonist of TNF (Tumor Necrosis Factor)-α, is the only approved biologic drug for moderate-to-severe HS, with a significant improvement ranging from 41.8% to 77% of treated patients, as shown in clinical trials and real-life observational studies [33]; the evidence of a “window of opportunity” supports the early use of adalimumab in HS to ensure better clinical response [34]. Surgical treatments (e.g., deroofing, incision and draining, local excision of the lesions) are taken into account in the most severe stage of HS with extensive and long-standing sinus tracts/fistulae formation or in treatment-refractory disease [35]. Smoking cessation, weight reduction, avoidance of triggers factors (friction, rubbing, sweating) in apocrine-bearing areas are accepted general measures to reduce exacerbation and ensure a better quality of life, especially in the milder forms of disease [35]. An extensive review on HS current treatment options and ongoing clinical trials has been recently published by Markota Čagalj et al. [33]; however, despite the great number of emerging treatments, the burden of the disease remains high, and long-lasting impairment of the patients’ quality of life has been reported [36]. Finally, no guidelines are currently available for different HS clinical phenotypes and its syndromic forms; thus, extensive, basic, and molecular research is still required to fully unravel the various mechanisms underlying this disease [36].

## 4. Genetic Landscape of HS

The pathophysiology of HS is complex and is determined by a crosstalk between environmental and lifestyle factors such as smoking and obesity [37]. A dysregulated innate and adaptive immune system with an up-regulation of pro-inflammatory cytokines/chemokines has been observed in HS patients and different variants in genes related to autoinflammation and keratinization pathways have been also reported [19,38]. However, the genetic scenario of HS in its different forms is still to be unraveled, as is considering recent growing evidence provided by the multiplicity of Next Generation Sequencing (NGS) tools and functional cell and organoids models for the validation of genetic variants. Here, we provide a lookout on the more recent findings related to the genetics of HS in the context of disease subtypes.

### 4.1. Genetics of Familial Cases of HS

Familial forms of HS have been reported in about 40% of cases (OMIM: # 142690 ACNE INVERSA, FAMILIAL, 1; ACNINV1 and # 613736 ACNE INVERSA, FAMILIAL, 2, ACNINV2) and an autosomal dominant mode of inheritance with an incomplete penetrance has been described, although the reported causative monogenic mutations are just explained by/associated with approximately 5% of familial HS cases [39,40]; recently, Dutch and Danish twin studies have reported an increase of around 80% in the rate of HS [40,41].

The first genetic study on HS was performed in 1984 by Fitzsimmons et al. [42] on three UK families, with a total of 21 HS patients (16 females and 5 males), suggesting that HS can be a single gene or Mendelian disorder with an autosomal dominant inheritance pattern of transmission, considering the familial aggregation and number of HS patients. One year later, the same authors [43] extended the study to other 23 families with a total of 62 HS patients (40 females and 22 males), finding in 11 families evidence in favour of a single gene Mendelian inheritance, as already previously observed; however, in nine families, no HS family history was observed at the time of survey. The same was found for another three families, in which no specific pattern of inheritance had been detected, possibly due, in both families, to HS misdiagnosis [43,44].

In 2000, J M Von Der Werth et al. [44] performed a re-examination of the familial cases identified 15 years earlier by Fitzsimmons at al. [42] with more accurate clinical characterization, confirming the concept that HS is a Mendelian disorder with an autosomal dominant inheritance pattern.

Only in 2006, M Gao et al. [45], in a HS Chinese four-generation family (nine HS patients, four females and five males) study, described a locus for HS located on chromosome 1p21.1–1q25.3, but no specific gene has been identified due to the considerable size of the genomic region. In 2010, B. Wang et al. [8] recognized *NCSTN* (Nicastrin) as a specific gene located precisely at 1q23.2 and subsequently, in all available HS familial patients, independent heterozygous loss-of-function mutations in three different genes *NCSTN*, *PSENEN* (Presenilin Enhancer, 19q13.12), and *PSEN1* (Presenilin 1, 14q24.2) have been found.

From this point on, the focus was mainly on these three genes that encode three of the four subunits of the gamma-secretase complex (GSC), even though some studies failed to reveal the causative role of gamma-secretase gene mutations in HS familial cases. In a U.K. study conducted by Pink et al. in 2011 [12], 53 individuals from seven multigenerational pedigrees were enrolled and the screening of *NCSTN*, *PSEN1*, and *PSENEN* genes did not reveal pathogenic variants in five out of the seven pedigrees examined; moreover, in a French study [11], only three out of fourteen pedigrees revealed the causative involvement of GSC genes, and similarly, mutational analysis of *NCSTN*, *PSEN1*, and *PSENEN* in twenty familial HS patients referred to a tertiary U.K. clinic, detected only two pathogenic variants in the coding region and splice site of *NCSTN* gene [13]. In a South Wales cohort of twelve familial HS probands, sequencing analysis of all genes encoding the gamma-secretase complex, including *PSEN2* (Presenilin 2), the homologue of *PSEN1*, as well as the two homologues *APH1A* (Aph-1 Homolog A), and *APH1B* (Aph-1 Homolog B) failed to reveal HS-associated mutations in these genes; most of the variants identified were intronic or synonymous substitutions without a functional impact on protein and correlation with the clinical phenotype. Taking together all the findings reported above, we could hypothesize that in several HS familial patients, the contribution of gamma-secretase genes is not pivotal or at least should be more deeply elucidated.

#### 4.1.1. Gamma-Secretase Complex Gene Mutations

GSC is a multi-subunit aspartyl protease complex that cleaves a series of integral type I transmembrane proteins; more than 90 proteins act as substrates and the most well-known are Notch and Amyloid precursor protein (APP). This process is known as regulated intramembrane proteolysis (RIP) that orchestrates biological signaling pathways by abolishing or promoting the effector molecules release [46]; an ever-increasing number of novel substrates made the gamma-secretase complex a key actor involved in several biological processes such as embryonic development and tissue homeostasis. As mentioned above, GSC consists of four subunit domains known as: (i) Nicastrin (NCSTN), (ii) Presenilin Enhancer 2 (PEN2), (iii) Presenilin 1 (PSEN1), and (iv) Anterior Pharynx Defective (APH) 1; moreover, another membrane protein called CD147, has been identified as non-essential regulator of the complex, whose absence increases GSC activity [47]; a GSC is highly heterogeneous due to the existence of two forms of presenilin (PSEN1 and 2) and two APH1 isoforms (APH1A and B) via alternative splicing, leading to at least six different possible gamma-secretase complexes with tissue- or cell type specificity [48].

The most widely reported genetic change occurring in familial HS is in the nicastrin-coding gene *NCSTN* (OMIM: # 142690 ACNE INVERSA, FAMILIAL, 1; ACNINV1) that encodes a 78 kDa type I single pass transmembrane protein, is highly glycosylated [49], and plays a crucial role in maintaining the stability of GSC and regulating intracellular protein trafficking [50]. It has been demonstrated that the blockage of NCSTN glycosylation leads to the insertion of immature NCSTN into the GSC leading to an altered trafficking of the mature complex [49]; moreover, NCSTN is also postulated to act as a gamma-secretase substrate receptor for the amino-terminal stubs generated by ectodomain shedding of type I transmembrane proteins [51].

The second most reported change in familial HS is in the Presenilin Enhancer 2 gene *PSENEN* (OMIM: # 613736 ACNE INVERSA, FAMILIAL, 2, ACNINV2). *PSENEN* encodes for a 101-amino acid integral membrane protein and acts as regulatory component of GSC [52]; biochemical studies have revealed that the sequence motif D-Y-L-S-F at the C-terminal end is crucial for binding to other GSC subunits whereas the length of the C-terminal tail is required for the formation of an active and functional GSC [53].

Presenilin 1 is composed of 9 transmembrane domains with an extracellular C-terminal end and a cytosolic N-terminus [54], that promotes proteolytic processing to make ~27–28 kDa N-terminal and ~16–17 kDa C-terminal fragments [55] and it has been observed that its overexpression led to the short-lived full-length protein accumulation in an inactive form [56].

Finally, APH1 is a 7 transmembrane helix protein expressed as two isoforms in two genetic loci (*APH1a* on chromosome 1 and *APH1b* on chromosome 15) and, via the alpha helix interaction motif glycine-X-X-X-glycine, is required for GSC proteolytic activity and for assembly the premature components [57].

NCSTN with PSEN1 and PSEN2 can establish a “secretasome” which allows for intramembranous proteolysis of the transmembrane proteins, including Notch [58].

Notch signaling is a highly conserved cell signaling that is crucial for cell–cell communication and multiple cell differentiation processes during both embryonic and adult life. Notch activation regulates skin homeostasis through growth arrest and cell terminal differentiation; indeed, Notch downregulates basal genes such integrins—allowing the spread of basal cells, promotes the expression of genes encoding early differentiation markers such as keratin 1 and involucrin, and prevents the expression of loricrin and filaggrin of the granular layer [59]; moreover, Notch regulates activity of both p21, which promotes cell cycle arrest in proliferating keratinocytes and p63, a master regulator of epidermal development promoting keratinocyte differentiation [60] and whose activity is counteracted by Notch [61].

Downregulation of Notch signaling pathways has been shown to alter keratinocyte differentiation program leading to an uncontrolled cell proliferation, as demonstrated in mouse models where Notch signaling alteration results in perturbation of sebaceous gland differentiation as well as terminal differentiation of the epidermis [62]; moreover, functional studies highlighted that the haploinsufficiency of genes encoding GSC results in defects in Notch signaling as the underlying mechanism, although potential additional genetic and epigenetic determinants could also contribute to [6], stating that both gamma-secretase complex and Notch signaling play a key role in the development of epidermal cysts and comedones, phenotypic traits of HS [63].

Several genetic studies on HS have thus focused mainly on Notch signaling alterations with a particular emphasis on the GSC; indeed, loss-of-function pathogenic variants in *NCSTN*, *PSEN1,* and *PSENEN* explain a subset of familial HS cases [13], leading it to be considered a single-gene disorders with an autosomal dominant inheritance pattern [9]. Conflicting findings concerning Notch signaling pathway involvement have also been reported; for example, PIK3R3 (Phosphoinositide-3-Kinase Regulatory Subunit 3) and AKT3 (AKT Serine/Threonine Kinase 3), two downstream signaling pathway components of NCSTN and Notch, have been found markedly overexpressed both in lesional and perilesional skin of HS patients when compared to control [64]; the levels of these proteins, in addition to NCSTN and Notch, were significantly higher in patients with mild disease when compared to those with moderate and severe HS. Additionally, functional studies demonstrated that several *NCSTN* missense variants associated with HS are functional and promote Notch signaling, counteracting the view that pathogenic variants in genes encoding gamma-secretase components cause disease simply as a result of haploinsufficiency that leads to impaired Notch signaling [10,65,66].

#### 4.1.2. Mutations in Genes Other Than the Gamma-Secretase Ones

Jifri et al. [18] reported two novel causal pathogenic variants in *MEFV* and *NOD2* genes in a two-generation HS family; the *MEFV* (MEditerraneanFeVer) gene encodes the protein pyrin, a member of the pyrin-domain (PYD)–containing proteins, mainly expressed in neutrophils and monocytes; the binding of pyrin to ASC (Apoptosis-Associated Speck-like protein containing a C-terminal caspase recruitment domain) leads to activation of ASC, with consequent recruitment and activation of procaspase-1. Active caspase 1 is crucial for the proteolytic activation of Interleukin (IL)-1β, which acts as the major cytokine in multisystem autoinflammatory disorders such as Familial Mediterranean Fever (FMF) [67]. Given that the frequency of *MEFV* mutations in HS patients is higher than that in healthy controls, it is likely that mutations of this gene may also contribute to the pathogenesis of HS [7,68]. *NOD2* (Nucleotide-binding oligomerization domain-containing protein 2) is an intracellular pattern recognition receptor (PRR) and member of the NOD-like receptor family that plays a crucial role in the immune response by activating the NF-κB (Nuclear actor kappa-light-chain-enhancer of activated B cells) protein [69]. *NOD2* sequence variants have been associated with Crohn’s disease and this gene is now considered an “IBD candidate gene” that can be categorized into microbial sensing to activate autophagy pathways [70]; more recently, *NOD2* has been also related to NOD2-associated autoinflammatory disease (NAID), a new entity characterized by infiltrated skin lesions, febrile recurrent episodes, arthritis, and gastrointestinal symptoms [71]. Collectively, these findings contribute to the evidence for a polygenic presentation mode for this disease, although further studies on familial HS are needed to highlight the pathophysiological mechanisms underlying this condition.

## 5. Genetics of Sporadic HS Cases

Despite the absence of fully penetrant variants, van Straalen et al. [41] suggested that sporadic forms of HS have a strong genetic background that contributes to their causality; moreover, environmental and epigenetic factors have been shown to contribute to HS susceptibility, supporting a multifactorial aetiology. To date, the nature of the genetic variants driving different forms of HS remains to be clarified since gamma-secretase gene mutations occur only in around 6% of sporadic HS [2].

Based on the increased evidence for a pivotal role of an unbalanced immune system in the HS pathogenesis, several genetic studies mainly focused on genes encoding for protein of the immune response. Savva et al. [72] investigated, in a cohort of 190 HS sporadic patients, single nucleotide polymorphisms (SNPs) in *TNF* (Tumor Necrosis Factor) and *TLR4* (Toll-like receptor 4) genes, finding that only one SNP (-238G/A, rs361525) of the promoter region of the *TNF* gene was associated with susceptibility to HS as well as with response to TNF antagonists, whereas statistical insignificance between *TLR4* variants and clinical features were found. Similarly, Giatrakos et al. [73] observed that SNPs in *IL-12Rb1* (Interleukin-12 receptor, beta 1), a subunit of the IL-12 receptor that binds both IL-12 and IL-23, did not play a significant role in genetic predisposition to HS; however, two common haplotypes—h1 and h2—have been recognized: carriers of h2 showed an increased risk of disease severity, whereas individuals with the h1 haplotype presented a late-onset disease. Subsequently, Giamarellos-Bourboulis et al. [74] demonstrated that high copy numbers of the β-Defensin Cluster conferred genetic susceptibility to HS, interfering directly with the clinical phenotype. Nine β-defensin genes, including *DEFB4* (Defensin Beta 4A) and *DEFB103* (Defensin Beta 103B), encoding for proinflammatory mediators human β-defensin-2 and human β-defensin-3, respectively, exist as a cluster presenting copy number variations; the same authors confirmed, in two independent cohorts, that the presence of more than six defensins cluster copy number had an increased risk of developing HS, whereas the presence of fewer than six copies was linked with early-onset disease, and fewer areas affected and less frequent presentation of permanent purulence of the skin lesions [74]. Finally, Agut-Busquet et al. [75] observed an association between MYD88 (Myeloid differentiation primary response gene 88),—a cytosolic adapter protein acting as a signal transducer in the IL-1 and TLR signaling pathways—,SNPs (for the GG genotype of rs6853), and susceptibility to severe HS.

## 6. Genetics of Syndromic HS Cases

In a subset of patients, HS can be a common manifestation of certain immune-mediated inflammatory diseases, presenting as syndromic HS [3]. HS syndromes in which genetic changes have been reported include PASH, PAPASH, and SAPHO syndromes; Braun-Falco et al. [76] first described two PASH families in which an increased number of CCCTG repeating in the promoter region of the *PSTPIP1* (Proline-Serine-Threonine Phosphatase Interacting Protein 1) gene was found. The functional effect of this genetic imbalance is controversial, since longer forms of the *PSTPIP1* gene have been also observed in patients with the aseptic abscess syndrome with or without Crohn’s disease, as well as in healthy subjects [77]. *PSTPIP1* encodes for a cytoskeleton-associated adaptor protein that interacts with several proteins, including pyrin, with which modulates several immunoregulatory functions; disease-causing mutations in this gene lead to a decreased inhibition of the inflammasome, with an up-regulation of caspase-1 and an increased inflammatory cytokines release—primarily IL-1β—which in turns triggers the release of proinflammatory markers producing a neutrophil-mediated autoinflammatory response [78]. After the first cases reported by Braun-Falco et al. [76], *PSTPIP1* mutations have been described in other PASH patients as well as in PAPASH probands [15,16,17]. Marzano et al. [19] demonstrated, in a case series of syndromic HS (5 PASH, 1 PAPASH and 1 PASH/SAPHO), the overexpression of cytokines/chemokines and molecules of the inflammatory network, along with genetic changes, confirming the link between the pathophysiology of HS and its syndromic forms and other autoinflammatory conditions. In this regard, pathogenic mutations in known autoinflammatory genes, including *NRPL3* (NLR family pyrin domain containing 3), have been observed in PASH patients [19]. NLRP3 is a key component of the innate immune system that acts as a pattern recognition receptor (PRR) by recognizing pathogen-associated molecular patterns (PAMPs) and danger-associated molecular patterns (DAMPs) [79]; it belongs to the NOD-like receptor (NLR) subfamily, and together with ASC forms a caspase-1 activating complex known as the NLRP3 inflammasome. Caspase-1 within the activated NLRP3 inflammasome complex in turn activates the inflammatory cytokine, IL-1β, the common denominator linking variants described in HS and its syndromic forms as well as in other autoinflammatory disorders [79,80]; the same authors also described pathogenic variants in other autoinflammatory genes including, among others, the *MEFV* gene in a PAPASH patient [19]. Similarly, a retrospective review including 109 complex HS patients, defined as having Hurley stage III (severe disease) and/or other inflammatory symptoms, revealed that 38% of them harboured *MEFV* pathogenic variants, suggesting that *MEFV* mutations may contribute to the pathogenesis of HS and its complex phenotypes [81]. The involvement of other autoinflammatory genes, including *NOD2*, *NLRC4* (NLR Family CARD Domain Containing 4), *WDR1* (WD Repeat Domain 1), *MPO* (Myeloperoxidase), and *OTULIN* (OTU Deubiquitinase With Linear Linkage Specificity), as well as *GJB2* (Gap Junction Protein Beta 2), a key gene of keratinization pathway, has been recently found by Marzano et al. In another cohort of syndromic HS patients (4 PASH, 3 PAPASH and 3 PASH/SAPHO), [7] corroborated the polygenic autoinflammatory nature of these HS syndromic forms.

## 7. Genetics of HS in the Setting of Other Diseases

A *GJB2* pathogenic variant in a PASH patient with gut inflammation has been described [7] and the same variation has been associated to Keratitis-Ichthyosis-Deafness (KID) syndrome, clinically characterized by keratitis, erythrokeratoderma, and neurosensory deafness; [82]; recently, four cases of KID occurring in association with the follicular occlusion triad—represented by HS, acne conglobata, and dissecting folliculitis of the scalp—have been reported [83]. The correlation between HS and *GJB2*—a member of the gap junction protein family with a key role in the growth, maturation, and stability of the epidermis [84]—is still to be clarified, but it has been speculated that HS could be the result of the hyperproliferative tendency of epidermis in KID patients to promote follicular plugging and cyst formation, promoting an exaggerated inflammatory response in resident immune cells [22,85].

Pathogenic variants in *NCSTN* and *PSENEN* genes have been described not only in HS familial and syndromic cases [86,87], as mentioned above, but also in HS-associated with Dowling–Degos disease (DDD) [65]; this latter is a rare autosomal dominant disorder of hyperpigmentation caused by loss-of-function mutations in the non-helical head domain of the keratin 5 (*KRT5*) gene [88]; other causative genetic changes include pathogenic variants in *POFUT1* (Protein O-Fucosyltransferase 1), *POGLUT1* (Protein O-Glucosyltransferase 1), and *PSENEN,* and all these genes may confer disease susceptibility by affecting Notch signalling pathways [14,25]. Interestingly, *POFUT1* and *POGLUT1* mutations have been associated to a clinical subtype of DDD that commonly involves non-flexural sites, whereas *PSENEN* and *NCSTN* pathogenic variants have been specifically attributed to a form of DDD associated with HS [14,25,65].

HS-like lesions have been also observed in nevoid acne and Nevus Comedonicus (NC), a rare epidermal nevus comprising of a group of dilated hair follicle openings filled with black keratinous plugs, that was initially described as “localized acne” [89]. In some cases of NC, a somatic missense mutation in *FGFR2* (Fibroblast Growth Factor-Receptor gene 2) gene has been identified; this gene is mainly expressed in keratinocytes, hair follicles, and sebaceous glands and is considered as a central player in the pathophysiology of acne vulgaris [90]. Higgins et al. [21] identified a rare heterozygous missense mutation in *FGFR2* in a patient with NC, acne, and additional features of HS, probably associated, not only with generalized comedones but also with clinical findings typical of HS.

Reports of HS patients and Dent disease 2 (DD2) suggested that genetic changes in the *OCRL1* (inositol polyphosphate-5-phosphatase 1) gene could be predisposing to HS [24]. OCRL1 is involved in the regulation of membrane trafficking and plays an important role in primary cilium formation; mutations in this gene cause, among other, DD2, a renal disease characterized by proximal renal tubular defect, hypercalciuria, nephrocalcinosis, and renal insufficiency [91]. Marzuillo et al. [24] described five DD2 patients, four of whom with concomitant HS and all these patients carried *OCRL1* mutations, resulting in significantly reduced inositol polyphosphate-5-phosphatase activity. Diminished OCRL1 protein results in increased phosphoinositol-4,5-bisphosphonate (PI (4,5) P2) that facilitates staphylococcal aggression in fibroblasts; therefore, the link between HS and DD2 could be an increased susceptibility to cutaneous infections, due to an accumulation of PI (4,5) P2 [24].

Two cases of HS occurring in concomitance with Darier’ disease have been recently reported; the authors suggested that a potential causal link between these two conditions may arise from an interaction between Notch homolog 1 and SERCA2, a calcium ATPase encoded by ATP2A2 (ATPase Sarcoplasmic/Endoplasmic Reticulum Ca2+), the gene mutated in Darier’ disease, also known as keratosis follicularis, an autosomal dominant skin disorder characterized by loss of adhesion between epidermal cells and abnormal keratinization [20].

Finally, a single case with HS and pachyonychia congenita, a genodermatosis associated with severe palmoplantar keratoderma (PPK) and dystrophic nails, has been reported by Pedraz et al. [23] and associated with a pathogenic variant in *KRT6A* (Keratin 6A) gene.

## 8. Conclusions

In this perspective on genetics of Hidradenitis suppurativa, we described the great genetic heterogeneity between sporadic, familial, and syndromic forms of HS. Only a minority of cases present mutations in gamma-secretase genes and when present, it could be insufficient to explain the clinical phenotype, suggesting that many genes/mutations not yet identified could contribute to HS susceptibility and disease-onset. Moreover, the crosstalk between clinical and epigenetic/environmental risk factors remains to be unraveled to better understand the complex pathogenesis of this disease and outline a clear etiology of each form of HS. In this regard, the implementation of integrated -omics studies are necessary to expand the knowledge on the exact pathophysiological nature of HS and its syndromic forms; GWAS (Genome-wide association study) could detect common genomic variants to HS, identifying new loci associated with susceptibility, disease severity, and response to treatment as well as exome/genome studies that would allow for the search of novel potential causative genes/mutations. However, we should keep in mind that GWAS studies on HS, due to the high statistical pressure, could miss important information about the biological pathways involved in the disease. In fact, the investigation should not be limited to single genetic variants since several studies failed to describe an HS common mechanism. Moreover, the weakness—if not absence—of an established genotype-phenotype correlation could be attributed to phenotypic heterogeneity that reflects pleiotropic genetic effects in overlap with modifiers gene and epigenetic modifications, and only an improved characterization of clinical features and consensus regarding phenotypic subtypes of HS will further assist with this.

We conclude our viewpoint emphasizing the strong need for an integrated approach using OMICs tools to explore the genome, epigenome, as well as the skin transcriptome of HS patients, together with a complete characterization in terms of clinical findings (including risk factors, comorbidities, family history), disease severity, type of treatments, and response to several drugs to better characterize the genetic and immunological basis of HS and thus unravel new potential targets for drug development.

## Figures and Tables

**Table 1:** Summary of genetic changes involved in all forms of HS.

Gene	Variant Segregation	Main Study Groups
NCSTN	Sporadic	Vural et al. 2021 [6]
	Syndromic HS	Marzano et al. 2022 [7]
	Familial	Wang et al. 2010 [8]; Wu et al. 2020 [9]; Xiao et al. 2016 [10]; Miskinyte et al.2012 [11]; Pink et al. 2011 [12]
PSENEN	Familial	Pink et al. 2012 [13]
	Familial syndromic HS	Pink et al. 2012 [13]
	HS + DDD	Ralser et al. 2017 [14]
PSTPIP1	Familial syndromic HS	Saito et al. 2018 [15]
	Sporadic syndromic HS	Marzano et al. 2013 [16]; Calderon-Castrat et al. 2016 [17]
MEFV	Familial	Jfri et al. 2020 [18]
	Sporadic syndromic HS + FMF	Marzano et al. 2014 [19]
NLRP3	Sporadic syndromic HS	Marzano et al. 2014 [19]
NOD2	Familial	Jfri et al. 2020 [18]
	Sporadic syndromic HS	Marzano et al. 2014 [19]
	Sporadic syndromic HS	Marzano et al. 2022 [7]
MPO	Sporadic syndromic HS	Marzano et al. 2022 [7]
NLRC4	Sporadic syndromic HS	Marzano et al. 2022 [7]
OTULIN	Sporadic syndromic HS	Marzano et al. 2022 [7]
ATP2A2	Darier’s disease + HS	Ornelas et al. 2016 [20]
FGFR2	Nevus Comedonicus + HS	Higgins et al. 2017 [21]
GJB2	Sporadic syndromic HS	Marzano et al. 2022 [7]
	Sporadic KID + HS	Maintz et al. 2005 [22]
IL1RN	Sporadic syndromic HS + FMF	Marzano et al. 2014 [19]
KRT6	Pachyonychia Congenita + HS	Pedraz et al. 2008 [23]
OCRL1	Dent disease + HS	Marzuillo et al. 2018 [24]
POFUT1	HS + DDD	Basmanav et al. 2014 [25]

HS, hidradenitis suppurativa; DDD, Dowling-Degos disease; FMF, familial Mediterranean fever; KID, keratitis-ichthyosis-deafness; NCSTN, nicastrin; PSENEN, presenilin enhancer; PSTPIP1 (proline-serine-threonine phosphatase interacting protein 1); MEFV, MEFV Innate Immunity Regulator, Pyrin; NLRP3, NLR Family Pyrin Domain Containing 3; NOD2, nucleotide binding oligomerization domain containing 2; MPO, myeloperoxidase; NLRC4, NLR Family CARD Domain Containing 4; Otulin, OUT deubiquitinase with linear linkage specificity; ATP2A2, ATPase sarcoplasmic/endoplasmic reticulum Ca2+ transporting 2; FGFR2, fibroblast growth factor receptor 2; GJB2, gap junction protein beta 2; IL1RN, interleukin 1 receptor antagonist; KRT6, keratin6A; OCRL1, inositol polyphosphate-5-phosphatase 1; POFUT1, protein o-fucosyltransferase 1.

## Data Availability

Not applicable.
